# Assessment of higher order cognitive skills in undergraduate education: modified essay or multiple choice questions? Research paper

**DOI:** 10.1186/1472-6920-7-49

**Published:** 2007-11-28

**Authors:** Edward J Palmer, Peter G Devitt

**Affiliations:** 1 Centre for Learning and Professional Development, University of Adelaide, Adelaide, Australia; 2 Dept of Surgery, University of Adelaide, Adelaide, Australia

## Abstract

**Background:**

Reliable and valid written tests of higher cognitive function are difficult to produce, particularly for the assessment of clinical problem solving. Modified Essay Questions (MEQs) are often used to assess these higher order abilities in preference to other forms of assessment, including multiple-choice questions (MCQs). MEQs often form a vital component of end-of-course assessments in higher education. It is not clear how effectively these questions assess higher order cognitive skills. This study was designed to assess the effectiveness of the MEQ to measure higher-order cognitive skills in an undergraduate institution.

**Methods:**

An analysis of multiple-choice questions and modified essay questions (MEQs) used for summative assessment in a clinical undergraduate curriculum was undertaken. A total of 50 MCQs and 139 stages of MEQs were examined, which came from three exams run over two years. The effectiveness of the questions was determined by two assessors and was defined by the questions ability to measure higher cognitive skills, as determined by a modification of Bloom's taxonomy, and its quality as determined by the presence of item writing flaws.

**Results:**

Over 50% of all of the MEQs tested factual recall. This was similar to the percentage of MCQs testing factual recall. The modified essay question failed in its role of consistently assessing higher cognitive skills whereas the MCQ frequently tested more than mere recall of knowledge.

**Conclusion:**

Construction of MEQs, which will assess higher order cognitive skills cannot be assumed to be a simple task. Well-constructed MCQs should be considered a satisfactory replacement for MEQs if the MEQs cannot be designed to adequately test higher order skills. Such MCQs are capable of withstanding the intellectual and statistical scrutiny imposed by a high stakes exit examination.

## Background

Problem-solving skills are an essential component of the medical practitioner's clinical ability and as such must be taught, learned and assessed during training. Entire curricula have been re-designed with this concept in mind. Problem-based learning is used in many teaching institutions and has its supporters and detractors. Despite criticism, it is undeniable that what problem-based learning sets out to achieve in terms of encouraging and developing the skills of synthesis, evaluation and problem-solving are valued components of a good medical education. In conjunction with the promotion of these skills, an effective assessment process is required. It has long been recognised that in the assessment of clinical competence problem-solving ability has been one of the most difficult areas to measure and quantify [1]. The modified essay question (MEQ) is one of several tools developed to try and assess this skill [2].

The MEQ is a compromise between the multiple-choice question (MCQ) and the essay. A well constructed MCQ will be unambiguous, clearly set to a defined standard and easy to mark (usually automatically), but more often than not tests little more than recall of fact [3]. An essay might test higher powers of reasoning and judgement but will be time-consuming to mark and risk considerable variation in standards of marking [4]. The MEQ is designed to sit in between these two test instruments in terms of the ability to test higher cognitive skills and the ease of marking to a consistent standard. The aim of the modified essay question is to broadly measure both the absolute amount of knowledge retained by the candidate and the ability of the candidate to use that knowledge to reason through and evaluate clinical problems. It accomplishes this by providing a clinical scenario with a number of steps. Progression through these stages should test the candidate's ability to understand, reason, evaluate and critique.

Construction of appropriate MEQs can be difficult [5] and a major criticism of this form of assessment is that MEQs often do little more than test the candidate's ability to recall a list of facts and frustrate the examiner with a large pile of papers to be hand-marked [6].

Although there is evidence to suggest that well constructed MEQs will test higher order cognitive skills [5], and that they can test different facets of understanding than MCQs [7], it is reasonable to ask if MEQ assessments in higher education are well constructed and if they are capable of assessing higher order cognitive skills. This paper describes such a study and is designed to gauge the effectiveness of the MEQ as a summative test tool in a clinical course. We have defined the effectiveness of the questions by their ability to measure higher cognitive skills, as determined by a modification of Bloom's taxonomy, and its quality as determined by the presence of item writing flaws.

## Methods

Fourth Year clinical students at the University of Adelaide underwent a written test as part of their overall assessment of performance for a nine-week surgical attachment. The same test instrument was used at the start of the attachment and on completion. The test material consisted of 50 MCQs and three MEQs (a total of 8 stages) and the questions were designed so that both types would cover similar test material. The content, focusing on core material, was matched in both the MCQ and the MEQ components of the examination. The MCQs had one correct answer and four distractors and were constructed to standard guidelines for MCQ construction [8,9].

In addition, the MEQ components of the Final MB BS examination papers for two consecutive years at the University of Adelaide were analysed. The first paper had 15 MEQs with a total of 68 stages, the other had 15 MEQs with a total of. 70 stages. The papers for each examination were assembled by one member of Faculty, who gathered contributions from individual clinicians. There was no formal instruction for the contributors on how to construct an MEQ, which would assess higher order cognitive skills, and the examination organiser undertook the final review of the submitted material.

In total, 33 MEQs made up of 146 stages were collected for analysis. The MEQs were written by at least 12 separate authors using the standard methodology for developing assessments within the faculty.

Each multiple-choice question was quantified independently as to its level of cognitive skill tested [10] and its structural validity [11] by two assessors. Each modified essay question and their individual components was also categorised independently by the two assessors according to the cognitive level measured by each question and its component parts. The assessors discussed their individual assessment and then produced a final grading for each MCQ and MEQ. The inter-rater agreement was calculated using Kappa statistics.

The data was classified using a modification of Bloom's hierarchy of cognitive learning [12,13]. Three levels were defined and classified as shown in Table 1. Level I, covered knowledge and recall of information, Level II covered comprehension and application, understanding and the ability to interpret data, and Level III tested problem-solving, the use of knowledge and understanding in new circumstances.

**Table 1:** Modified Bloom's taxonomy

Level I:	Knowledge
	-recall of information
Level II:	comprehension and application
	-understanding and being able to interpret data
Level III:	problem-solving
	-use of knowledge and understanding in new circumstances.

The rating scale shown in Table 2 was used to judge the rigor of the multiple-choice questions according to the presence of any item-writing flaws.

**Table 2:** Rating scale used to judge the rigor of the multiple-choice questions according to the presence of any item-writing flaws.

Rating	Conditions required to achieve rating
1.	Pass the cover test and no item-writing flaws
2.	Pass the cover test and 1 to 2 item-writing flaws
3.	Cover test dubious and no item-writing flaws
4.	Fail the cover test and 1 to 2 item-writing flaws
5.	Fail the cover test and more than 2 item-writing flaws

The item-writing flaws were defined as:

• Repetition of part of the stem in an option

• Use of qualifiers within an option

• Complicated or ambiguous stem

• Negative questions not clearly stated

• Use of double negatives

• Absolute options (e.g., never, always, all-of-the-above)

The cover test has been defined as the ability to surmise the answer from the stem of an item alone, with the correct answer and the distractors covered up [9].

## Results

Table 3 illustrates an example of the coding of 2 MCQs. Neither of the MCQs in this table displayed item-writing flaws. Item 1 in the table was judged to be testing lower order cognitive skills than item 2.

**Table 3:** Sample coding of MCQs

Question	Modified Bloom's taxonomy categorisation	Explanation
A 16 year old obese schoolgirl is admitted with acute pancreatitis. The most likely underlying cause would be	1	This question is a test of knowledge recall only.
A. familial.		
B. hyperparathyroidism.		
C. alcohol.		
D. gallstones.		
E. trauma.		
8. A 68-year-old man is hospitalised with his third attack of acute cholecystitis in two years. He is started on a course of antibiotics. He suffered a myocardial infarction one month ago. An isotope scan performed six weeks prior to his present illness showed a non-functioning gallbladder. Which one of the following is the most appropriate treatment?	3	There is assumed knowledge in this question. The student needs to make a judgement and evaluation to choose the most appropriate management option.
A. immediate percutaneous cholecystolithotomy.		
B. start on chenodeoxycholic acid.		
C. allow patient to settle and then perform cholecystectomy within 48 hours.		
D. allow patient to recover and delay surgery for 5 months.		
E. proceed to immediate cholecystectomy.		

Table 4 illustrates stages of an MEQ requiring different levels of cognitive skill to answer. The first two items in the table come from the same MEQ. The last item was obtained from a different question.

**Table 4:** Sample coding of MEQs

Question	Modified Bloom's taxonomy categorisation	Explanation
A 46 year old woman presents to the emergency department with a three month history of early satiety and anorexia. Over the last two weeks she has been vomiting most days and has been unable to eat or drink much over the last few days. Describe what other information you would seek from the history that would help you establish a diagnosis and justify your answers.	3	Knowledge recall is required, but there is significant interpretation of data required. This makes this a Bloom level 2 at minimum. However, there is a need to evaluate other data, not provided explicitly in this problem in order to arrive a t a diagnosis (problem solving skills). This makes this question a Bloom level 3.
From the history you think that the patient has gastric outlet obstruction. Describe the physical findings you would look for on examination and explain why they might occur.	2	Knowledge recall is required but the student requires understanding of a number of different processes to answer the question correctly. There is no problem solving required, thus making this a Bloom level 2 question.
<from a different problem> Assuming that a mammogram was to be performed as part of the work-up, what are the features suggesting malignancy that would be sought?	1	Knowledge recall of features of malignancy. Requires no understanding of the overall problem.

The assessors showed a close correlation in their assessment of the questions according to the modified Bloom's taxonomy categorisation. The reliability between the two assessors and the final mark was good with values of Kappa equal to 0.7 and 0.8 for the MCQs and 0.7 and 0.8 for the MEQs.

**Figure 1:**
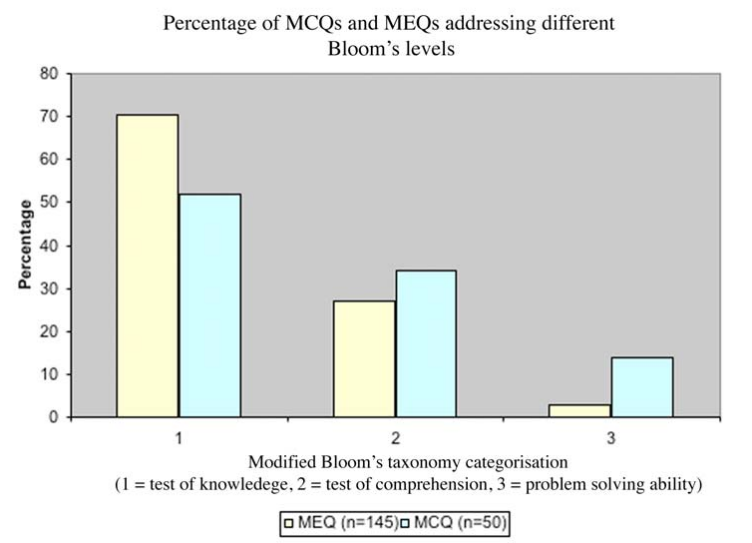
Percentage of MCQs and MEQs addressing different Bloom's levels of cognitive skills.

The overall performances of the MCQs and the MEQs were compared for their ability to test higher cognitive skills (Figure 1). Just over 50% of the MCQs in the Fourth Year examination paper focussed only on recall of knowledge and the largest proportion of MEQs also focussed on this low level cognitive skill. A similar proportion of MCQs and MEQs tested middle order cognitive skills and, rather surprisingly, MCQs were better at addressing the highest order cognitive skills compared with MEQs.

Each of the Final Examination papers for 2005 and 2006 contained 15 MEQs and there were a total of 68 and 70 sections respectively (average 4.5 and 4.7 sections per question). In the 2005 paper 51% of the questions tested factual recall (Bloom level I), 47% tested data interpretation (Bloom level II) and only 2% tested critical evaluation. The pattern was similar for the 2006 paper with 54% testing Bloom level I cognitive skills and the remainder (46%) testing Bloom level II.

**Figure 2:**
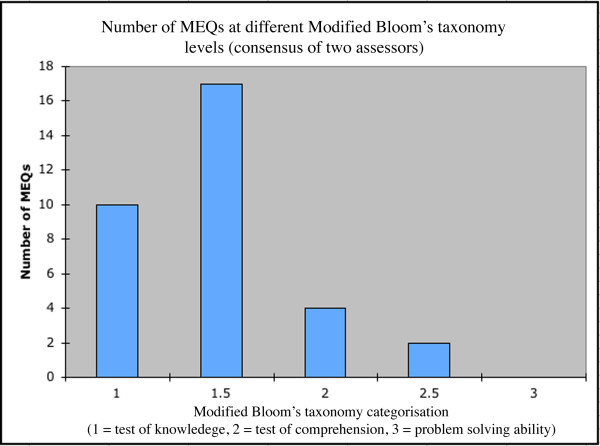
Number of MEQs at different Modified Bloom's taxonomy levels (consensus of two assessors).

The 33 MEQs had an average Bloom categorisation of 1.35 with a standard deviation of 0.4. The distribution is shown in Figure 2.

The assessors showed a close correlation in their assessment of the multiple-choice questions according to the item writing flaws categorisation. The reliability between the two assessors and the final mark was moderate, with Kappa equal to 0.5 and 0.6.

An analysis of the structural validity of the MCQs showed that 80% passed the cover test and contained no item-writing flaws. Twenty percent of questions were flawed, but most of these flaws were only of a minor nature and only one question out of the fifty was sufficiently flawed to call into question its structural validity.

## Discussion

For an assessment to be effective, there are a number of issues to be considered. Resource considerations are important, and this may have some impact on the style of exam chosen. True-false, multiple-choice and extended matching questions can be marked automatically and may have a relatively low impact on academic time, compared to the marking of MEQ and essay questions. Based on resource considerations alone, MEQs may be considered an inferior form of assessment, but there are other issues, which must be considered.

The reliability and validity of an assessment is vitally important. A reliable assessment will provide consistent results if applied to equivalent cohorts of students. MCQs benefit from a high reliability when the set of questions is valid and there are sufficient numbers of questions, as do True-False questions [14]. MEQs and standard essay questions can have good reliability provided multiple markers are used. Validity of content should always be carried out regardless of the type of assessment tool used. At a minimum this should include content validity and construct validity. Other measures of validity such as concurrent and predictive validity are also relevant but can be far more challenging to determine. The ability of assessments to discriminate effectively between good and poor candidates, as well as the fidelity of the assessment are also important considerations in evaluating an assessment tool.

We have shown that in a standard mid-course multiple-choice examination paper a substantial component of that examination will focus on testing higher cognitive skills. Yet conversely and perversely, in an examination specifically designed as part of the exit assessment process a disproportionately high percentage of modified essay questions did little more than measure the candidates' ability to recall and write lists of facts. This may be inappropriate when it is considered that the next step for most of the examinees is a world where problem-solving skills are of paramount importance. The analysis has shown that it is possible to produce an MCQ paper that tests a broad spectrum of a curriculum, measures a range of cognitive skills and does so, on the basis of structurally sound questions. It is important to recognise that these results are from one institution only, and the processes used to design assessments may not be typical of other institutions. The generalizability of the results is also worth considering. In this study there were many authors involved in writing the questions. Although it was not possible to isolate individual authors, at least a dozen individuals were involved, and there was little variation in the overall Bloom categorization of the MEQs. This suggests that the findings of this study may be transferable to other schools.

The apparent structural failure of the MEQ papers was not likely the result of a conscious design decision on the part of those who wrote the questions, but may have been a lack of appreciation of what an MEQ is designed to test. This resulted in a substantial proportion of the questions measuring nothing more than the candidates' ability to recall and list facts.

This relatively poor performance of MEQs has been observed by others. Feletti [15] reported using the MEQ as a test instrument in a problem-based curricula. In their study the percentage of the examination that tested factual recall varied between 11% and 20%. The components testing problem-solving skills ranged from 32% to 45%. That the proportion of factual recall questions in the current study was higher than that observed by Feletti might well reflect a lack of peer-review when the examination was set. The Feletti data showed that as the number of items increased in the examination, the ability to test cognitive skills, other than factual recall, fell. In other words, the shorter the time available to answer an item, the more likely the material would focus on recall of fact. The University of Adelaide papers allowed 12 minutes a question or less than 3 minutes per stage. This is considerably less than the 2 – 20 minutes per item in the Feletti study.

The open-ended question has low reliability [15] and an examination based on this format is unable to sample broadly. The essay has only moderate inter-rater reliability for the total scores in free-text marking and low reliability for a single problem [16]. Such an examination is also expensive to produce and score, particularly when measured against a clinician's time. It makes little sense to use this type of assessment to test factual knowledge, which can be done much more effectively and efficiently with the MCQ.

Our study has confirmed the impressions reported by others that MEQs tend to test knowledge as much as they measure higher cognitive skills [5]. If an MEQ is to be used to its full value it should present a clinical problem and examine how the students sets about dealing with the situation with the step-wise inclusion of more data to be analysed and evaluated. Superficially, this is what the MEQs in this study set out to do, but when the questions were examined closely, most failed and did no more than ask the candidates to produce a list of facts.

The present study has shown that it is possible to construct a multiple-choice examination paper, which tests those cognitive skills for which the MEQ is supposedly the instrument of choice. These observations raises the question of why it is necessary to have MEQs at all, but the potential dangers of replacing MEQs with MCQs must be considered.

It is generally thought that MCQs focus on knowledge recall and MEQs test the higher cognitive skills. When the content of both assessments is matched the MCQ will correlate well with the MEQ and the former can accurately predict clinical performance [2]. This undoubtedly relies upon a well-written MCQ designed to measure more than knowledge recall.

A good MCQ is difficult to write. Many will contain item writing flaws and most will do no more than test factual recall. Our study has shown that this does not necessarily have to be the case, but it cannot be assumed that anyone can write a quality MCQ unaided and without peer review.

If MCQs are to be used to replace MEQs or similar open-ended format, the issue of cueing must be considered. The effect of cueing is usually positive and can lead to a higher mean score [17]. Conventional MCQs have a cueing effect which has been reported as giving an 11-point advantage compared with open-ended questions. It has been shown that if open-ended questions do not add to the information gained from an MCQ, this difference in the mean score may not matter, particularly if it can lead to the use of a well structured MCQ testing a broad spectrum of material with an appropriate range of cognitive testing [18]. Grading could be adjusted to take into account the benefits of cueing.

Other options to improve the testing abilities of the MCQ type of format is to use extended matching questions and uncued questions [19]. These have been put forward as advances on the MCQ, but these test formats can be easily misused with the result that they may end up focusing only on knowledge recall [4,19,20].

The criticisms levelled at MCQs are more a judgement of poor construction [11,21] and the present study suggests that a similar criticism should be levelled at MEQs. We would go further, and suggest that assessment with well-written MCQs has more value (in terms of broad sampling of a curriculum and statistical validity of the test instrument) than a casually produced MEQ assessment. This is not suggest that MEQs should never be used, as they do have the capability to measure higher cognitive skills effectively [5], and there is evidence to suggest that MEQs do measure some facets of problem solving that an MCQ might not [7].

The measurement of problem-solving skills is important in medicine. MEQs seem ideally suited for this process, but it is possible to use a combination of MEQs and MCQs in a sequential problem solving process, where the ability to solve problems can be separated to some extent from the ability to retain facts [22]. The computer may be the ideal format for this, and there are examples of problem solving exercises using the electronic format readily available [23].

When designing an assessment, which may consist of MCQs or MEQs, it is important to recognise the potential strengths of both formats. This study has shown that if an MEQ is going to be used to assess higher order cognitive skills, there needs to be a process in place where adequate instruction is given to the MEQ authors. If this instruction is not available, and the authors can construct high quality MCQs, the assessment may be better served by containing more MCQs than MEQs. The reduced effort in marking such an assessment would be of benefit to faculties struggling with limited resources.

## Conclusion

Apart from its ability to assess appropriate cognitive skills, any assessment instrument should be able to withstand the scrutiny of content and construct validity, reliability, fidelity and at the same time discriminate the performance levels of the cohort being tested. We suggest that a well-constructed peer-reviewed multiple-choice question meets many of the educational requirements and advocate that this format be considered seriously when assessing students. Benefits of automated marking, and potentially high reliability at low cost make MCQs a viable option when writing high stakes assessments in clinical medicine.

## Competing interests

The author(s) declare that they have no competing interests.

## Authors' contributions

PGD conceived of the study. EP and PGD designed, coordinated and carried out the study. EP carried out the statistical analysis. Both authors participated in the manuscript and read and approved the final version.

## Pre-publication history

The pre-publication history for this paper can be accessed here:


